# The importance of appropriate diagnostics in prosthetic joint infection: letter to the editor of BMC musculoskeletal disorders

**DOI:** 10.1186/s12891-021-04117-8

**Published:** 2021-03-08

**Authors:** Martin McNally, Marjan Wouthuyzen-Bakker, Ricardo Sousa, Bridget Atkins, Alex Soriano

**Affiliations:** 1 The Bone Infection Unit, Nuffield Orthopaedic Centre, Oxford University Hospitals, Oxford, UK; 2 Department of Medical Microbiology and Infection Prevention, University of Groningen, University Medical Center Groningen, Groningen, Netherlands; 3 Porto Bone Infection Group (GRIP), Orthopaedic Department, Centro Hospitalar Universitário do Porto, Porto, Portugal; 4 Head of Infectious Diseases Department, Hospital Clinic of Barcelona, Barcelona, Spain

**Keywords:** Prosthetic joint infection, Diagnosis, Histology, Synovial fluid culture

## Abstract

Assessment of a new diagnostic test must be performed against an acceptable and validated standard to allow comparison with other studies. We are concerned that the adoption of lower diagnostic criteria in this paper has contributed to an over-diagnosis of prosthetic joint infection and makes interpretation of the results difficult.

## Main text

We read with interest the paper from Morgenstern et al. (2020) [1] reporting on microcalorimetry in the diagnosis of prosthetic joint infection (PJI). This is a novel and promising technique which merits assessment in this important clinical indication. However, we are concerned about the ‘gold standard’ used to diagnose PJI and the misleading referencing in this study.

The paper reports 107 hip and knee cases, but only 69 (65%) had surgery, and of these, only 61(57%) had tissue culture, 48(45%) had histological analysis and 28(26%) had sonication cultures. The others had diagnosis defined largely on preoperative synovial fluid analysis.

In assessing a new test, it is essential to use a robust definition of PJI which has been validated. This study uses an unvalidated definition which they describe as their ‘institutional criteria’. PJI was diagnosed with a synovial leukocyte count > 2000 cells/μl or > 70% granulocytes or positive histology with ≥2 granulocytes/high power field. These figures are supported with 9 references but 6 are from the same author group and provide no validation data for these cut-offs. The only fully independent reference [2] concluded that > 4200 and > 80% was appropriate in PJI of the hip, possibly reducing to > 3000 only if serum C-reactive protein was included in the definition.

Similarly, the quoted reference for histology [3] describes ≥23 granulocytes per 10 high power fields; not ≥2 per single field. It should be noted that 2 cells in 1 field is not similar to 23 cells over 10 fields. In aseptic loosening, it is much easier to find one piece of tissue which has some minor inflammation (1 or 2 granulocytes) due to mechanical irritation. Finding 23 granulocytes across multiple fields is much more indicative of infection. Therefore, choosing 2 granulocytes in one field is a much lower threshold than 23 in 10 and cannot be regarded as similar just because 2 in 1 is mathematically close to the average of 23 in 10.

This is important as all the parameters have been reduced, making overdiagnosis of PJI more likely, particularly when tissue culture is negative or not performed, as was common in this study. It is not appropriate to simply claim increased sensitivity of diagnosis in this way. Increased sensitivity is almost always achieved by reduction in specificity. In two previous studies [4, 5] the 2000 cells/μl cut-off gave specificities of 89 and 75% respectively.

The study confirms this bias with its very high culture negative rate. PJI is caused by the presence of bacteria in the joint. In patients diagnosed with PJI, only 45% (15/33) of the tissue cultures performed grew bacteria, which is one of the lowest reported rates in the literature. Only 39% (18/46) of synovial fluid cultures were positive and only 59% (10/17) of sonicate fluids, in patients claimed to have PJI. This is surprising as this same group has repeatedly published that sonication will identify around 80% of organisms in PJI, even in patients receiving antibiotics. In their previous study [6], they claimed that 21% of infections could be missed by the older definitions of PJI. In this new study, we are told that between 55 and 61% are being missed by accepted bacteriological protocols, from a laboratory which specializes in bone and joint infection. We believe that this supports our view that the combination of reduced tissue diagnoses, lower cell counts, lower histology criteria and missing investigations all contribute to overdiagnosis of PJI.

The authors regard preoperative histology as ‘not feasible’ quoting two references for this statement [7, 8] which do not mention preoperative histology at all. This ignores preoperative percutaneous needle synovial biopsy which is well studied.

This study is at best misleading and gives the impression that it is reporting a validated and accepted PJI definition. The lowering of diagnostic cut-offs to arbitrary levels and the low percentage of intraoperative sampling makes it impossible to fairly evaluate microcalorimetry or to compare this method with other diagnostic tests. Perhaps publication of the raw data for each patient would help elucidate the actual rate of PJI in the study and the efficacy of microcalorimetry.

Yours sincerely,

**Professor Martin McNally**, The Bone Infection Unit, Nuffield Orthopaedic Centre, Oxford University Hospitals, Oxford, UK.

**Dr Marjan Wouthuyzen-Bakker**, Department of Medical Microbiology and Infection Prevention, University of Groningen, University Medical Center Groningen, Netherlands.

**Professor Ricardo Sousa**, Porto Bone Infection Group (GRIP), Orthopaedic Department, Centro Hospitalar Universitário do Porto, Portugal.

**Dr Bridget Atkins**, The Bone Infection Unit, Nuffield Orthopaedic Centre, Oxford University Hospitals, Oxford, UK.

**Professor Alex Soriano**, Head of Infectious Diseases Department, Hospital Clinic of Barcelona, Spain.

## Data Availability

N/R

## Abbreviation

PJI: Prosthetic joint infection
